# Adenocarcinoma masked by re‐expansion pulmonary edema after chest drainage for pneumothorax

**DOI:** 10.1111/1759-7714.13136

**Published:** 2019-07-09

**Authors:** Jae Jun Jung, Hyo Jung An, Kyung Nyeo Jeon, Jong Woo Kim

**Affiliations:** ^1^ Department of Thoracic and Cardiovascular Surgery College of Medicine and Institute of Health Science, Jinju, Gyeongsang National University Changwon Hospital Changwon South Korea; ^2^ Department of Pathology Gyeongsang National University Changwon Hospital Changwon South Korea; ^3^ Department of Diagnostic Radiology College of Medicine and Institute of Health Science, Jinju, Gyeongsang National University Changwon Hospital Changwon South Korea

**Keywords:** Adenocarcinoma, computed tomography, ground‐glass opacity, pneumothorax, re‐expansion pulmonary edema

## Abstract

Re‐expansion pulmonary edema is a rare complication that may occur after chest drainage performed for pneumothorax. This condition manifests as areas of ground‐glass opacities (GGO) and septal thickening on imaging studies. In the case reported here, chest computed tomography (CT) showed diffuse ground‐glass opacity secondary to ruptured bullae in a patient who underwent chest tube drainage for pneumothorax, suggesting re‐expansion pulmonary edema. Histopathological examination of lung tissue resected from the vicinity of the bullae showed focal adenocarcinoma, which was masked by re‐expansion pulmonary edema on preoperative computed tomography. Right upper lobectomy with mediastinal lymph node dissection was performed on postoperative day 3.

## Introduction

The incidence of re‐expansion pulmonary edema after chest drainage in patients with pneumothorax is 14%–17%.^1^ The radiologic findings of re‐expansion pulmonary edema include ground‐glass opacity (GGO), septal thickening, focal consolidation, and atelectasis.^2^ Diffuse GGO in these patients may mask pulmonary adenocarcinoma. Therefore, it is very important to rule out pulmonary adenocarcinoma in patients with re‐expansion pulmonary edema.

## Case Report

**Figure 1:**
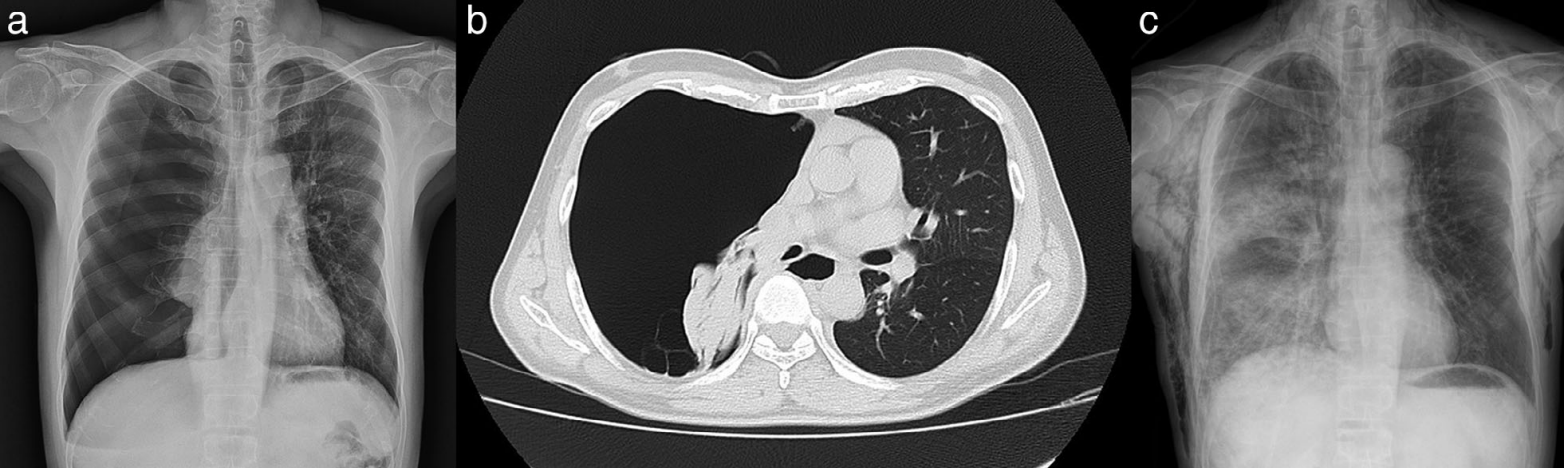
(**a**) Chest radiograph showed right‐sided pneumothorax, **(b**) Unenhanced chest CT showed a completely collapsed right lung, (**c**) Chest radiograph obtained after chest tube insertion showed diffuse consolidation and GGO, suggesting re‐expansion pulmonary edema. Subcutaneous emphysema is visualized. CT, computed tomography; GGO, ground‐glass opacity.

A 49‐year‐old man presented to another hospital complaining of chest pain and dyspnea. Chest radiography and computed tomography (CT) revealed right‐sided pneumothorax with lung collapse (Fig 1a,b). The patient's dyspnea worsened after the insertion of a 24‐Fr chest tube, and he was transferred to our hospital. He had a negative history of trauma or malignancy, and laboratory investigations revealed no abnormalities. Chest radiography performed at our hospital revealed diffuse consolidation and GGO in the right lung, suggesting re‐expansion pulmonary edema (Fig 1c).

**Figure 2:**
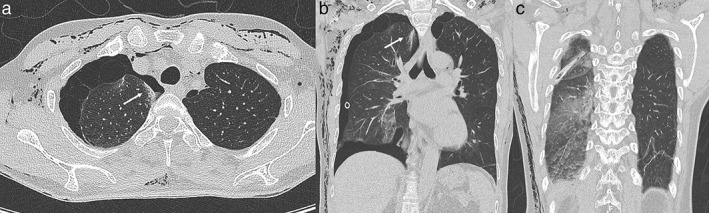
(**a**) Chest CT scan (coronal view) showed apical bullae and patchy areas of GGO in the right lower lobe. Another focal area of GGO is observed in the medial aspect of the right upper lobe (white arrow), (**b**) Chest CT scan (axial view) showed a clear view of multiple bullae and a focal area of GGO in the right upper lobe (white arrow), (**c**) Chest CT scan (coronal view) showed re‐expansion pulmonary edema in the right posterior lung zone manifesting with diffuse GGO and interlobular septal thickening. CT, computed tomography; GGO, ground‐glass opacity.

The patient received diuretic therapy and lung care for five days after admission. A large quantity of air leakage occurred through the chest tube. Preoperative chest CT showed multiple bullae in the apical area of the right upper lobe (RUL), as well as diffuse areas of GGO, and interlobular septal thickening in the right posterior lung zone, suggesting unresolved re‐expansion pulmonary edema (Fig 2a‐c). Focal GGO observed in the medial aspect of the RUL were attributed to the re‐expansion pulmonary edema at that time (Fig 2a,b).

**Figure 3:**
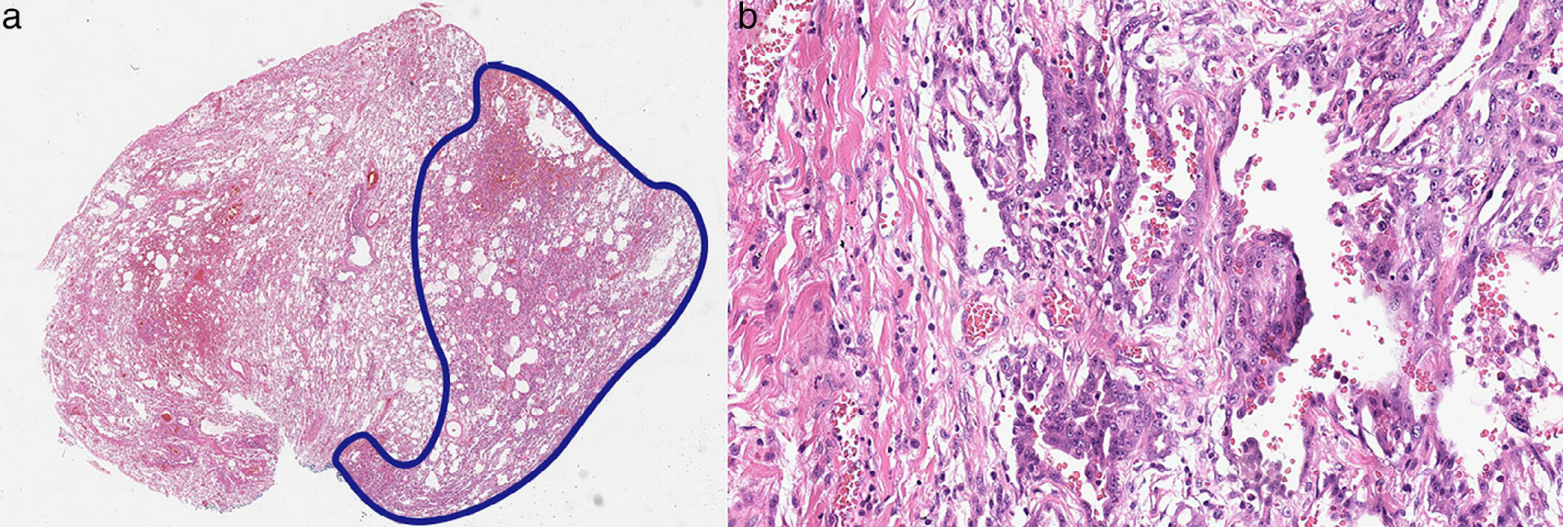
(**a**) Apart from the subpleural bullae, subpleural adenocarcinoma tumor is seen on the right in this section. The boundary of adenocarcinoma is marked with a blue line (hematoxylin eosin stain, ×10), (**b**) Light microscopy (high magnification) indicated a small area of acinar glands infiltrating the fibrous stroma. The invasive component measures <5 mm and suggests minimally invasive adenocarcinoma (hematoxylin eosin stain, ×200).

Elective right‐sided video‐assisted thoracoscopic surgery (VATS) was performed for wedge resection. The patient was positioned laterally after intubation. Wedge resection via VATS was performed to treat multiple large‐sized bullae in the RUL. The patient remained stable intraoperatively, was transferred to the recovery room, and showed an uneventful postoperative course. Histopathological examination of the resected specimen revealed adenocarcinoma in the subpleural area, apart from the subpleural bullae (Fig 3a). The maximum diameter of the tumor was 2.1 cm, and the diameter of the abutment with the stapler line was 0.3 cm. The adenocarcinoma demonstrated a lepidic growth pattern with a small (<5 mm) invasive component, suggesting minimally invasive adenocarcinoma (Fig 3b). The Ki‐67 labeling index was significantly higher than that in the non‐tumorous lung parenchyma. Right upper lobectomy with mediastinal lymph node dissection via VATS was performed the following day. Following lobectomy, there was residual tumor visible on histologic examination, with maximum diameter of 1.0 cm. In addition, there was edematous fluid within the alveoli, and congestion in the alveolar capillaries, possibly due to re‐expansion pulmonary edema. Final pathological staging confirmed pT1bN0M0 adenocarcinoma.

## Discussion

Re‐expansion pulmonary edema, a rare finding occurring following chest tube drainage performed for pneumothorax, has a mortality rate of 20%.^3^ The contributory pathophysiological mechanisms are increased permeability of injured pulmonary vasculature and injury to the vascular endothelium secondary to reperfusion injury.^4^, ^5^ In our case, we treated re‐expansion pulmonary edema for five days after chest drainage. Preoperative chest CT confirmed re‐expansion pulmonary edema and large bullae in the pneumothorax site, and no other radiologic findings were observed. Adenocarcinoma was diagnosed by histopathological examination of the wedge resection specimen. We performed right upper lobectomy with mediastinal lymph node dissection on day 3 after wedge resection. This case highlights that GGO which occurs in cases of re‐expansion pulmonary edema is often indistinguishable from the GGO associated with adenocarcinoma, due to which, pulmonary adenocarcinoma may be masked by re‐expansion pulmonary edema.

In summary, the GGO pattern of re‐expansion pulmonary edema and adenocarcinoma are difficult to distinguish. Therefore, in such patients, if the re‐expansion pulmonary edema has sufficiently resolved then surgery can proceed. If surgery is to be performed early, chest CT should be checked shortly postoperatively.

## Disclosure

The authors report no conflicts of interest.
